# Genome-Wide Identification and Expression of Neuropeptides and Their Expression Patterns After RNAi of CHH Genes in Pacific White Shrimp *Litopenaeus vannamei*

**DOI:** 10.3390/biology13121038

**Published:** 2024-12-11

**Authors:** Long Zhang, Lichao Sun, Guanghao Song, Beibei Wang, Yanting Cui, Fei Liu, Yuquan Li, Zhongkai Wang

**Affiliations:** School of Marine Science and Engineering, Qingdao Agricultural University, Qingdao 266109, China

**Keywords:** neuropeptides, gene expansion, alternative splicing, CHH, VIH

## Abstract

Neuropeptides are pivotal in regulating a broad spectrum of developmental, physiological, and behavioral processes throughout the life cycle of crustaceans. In this comprehensive study, we utilized a multiomics approach to characterize neuropeptide precursors and to assess the expression profiles of neuropeptide-encoding genes across various tissues and developmental stages in the Pacific white shrimp, *Litopenaeus vannamei*. Additionally, we explored the differential expression of neuropeptide genes in the eyestalk before and after the RNA interference-mediated suppression of crustacean hyperglycemic hormone (CHH) and vitellogenesis-inhibiting hormone (VIH) gene expression. Our study identified a total of 125 neuropeptide-encoding genes in *L. vannamei*, with 54 of these genes previously uncharacterized in the genome. Notably, certain neuropeptide-encoding gene families showed significant expansion, as demonstrated by the discovery of 10 adipokinetic hormone/corazonin-like peptide (ACP) genes, 55 CHH superfamily genes, and 13 pigment-dispersing hormone (PDH) genes. Alternative splicing was also found to play a crucial role in generating functionally diverse neuropeptides; for example, the agatoxin and calcitonin genes undergo alternative splicing that leads to the production of three distinct agatoxin neuropeptides and two distinct calcitonin neuropeptides, respectively. Neuropeptide genes are predominantly expressed in neuroendocrine tissues, including the eyestalk, cerebral ganglia, thoracic ganglia, and ventral ganglia. During the embryonic development of *L. vannamei*, with the exception of the molt-inhibiting hormone (MIH) gene, all monitored genes display minimal expression from the zygote stage through to the larval in membrane (Lim) stage. In contrast, the majority of these genes exhibit a steady uptick in expression from the nauplius stage onwards, culminating in the post-larval stage. Furthermore, comparative transcriptomic analysis of the eyestalk revealed that the expression of the majority of neuropeptide genes was downregulated following the suppression of CHH and VIH gene expression. This downregulation was significantly associated with the enrichment of pathways related to amino acid metabolism and hormone synthesis. The findings of this study provide valuable insights for future research aimed at elucidating the role of neuropeptides in regulating physiological functions in *L. vannamei*, potentially leading to advancements in shrimp aquaculture practices.

## Simple Summary

Neuropeptides are small molecules that play key roles in regulating various developmental, physiological, and behavioral processes in crustaceans. In this study, we focused on the Pacific white shrimp, *Litopenaeus vannamei,* to identify and understand the genes responsible for producing these neuropeptides. Using advanced genetic analysis techniques, we discovered 125 genes that encode neuropeptides, with 54 of them being newly identified. We found that these genes are mainly active in the shrimp’s nervous system, particularly in the eyestalk, brain, and nerve ganglia. Additionally, we explored how silencing two specific genes, CHH and VIH, which are important for hormone regulation, affected the expression of other neuropeptide genes. Our results showed that silencing these genes led to a significant decrease in the activity of many neuropeptide genes, affecting pathways related to metabolism and hormone production. This research provides valuable insights into the genetic mechanisms that control shrimp growth and development, which could help improve shrimp farming practices by improving growth rates and disease resistance.

## 1. Introduction

Neuropeptides are key signaling molecules that play an indispensable role in orchestrating a diverse array of developmental, physiological, and behavioral processes throughout the life cycle of crustaceans [1]. These molecules are directly encoded in the genome and are initially translated as large precursor molecules, called pre-prohormones, which require further post-translational processing for their biological activity and stability [2]. The mature neuropeptides are typically short peptides composed of 3–100 amino acid residues and are involved in a wide range of physiological processes, including reproduction, metabolism, growth, and locomotion [3,4]. Due to their key roles in these fundamental processes, neuropeptides are often targeted for modulating these processes to align with market demands in commercially important species.

The Pacific white shrimp, *Litopenaeus vannamei*, has gained global recognition as the most crucial species for shrimp aquaculture [5]. Its production has faced challenges due to environmental stressors and disease exposure [6,7,8]. Neuropeptides, which are pivotal in regulating a range of physiological and behavioral processes in crustaceans, offer substantial potential for enhancing aquaculture practices. Gaining insights into the genetic mechanisms that govern growth, development, and stress response in shrimp can lead to strategies that boost growth rates, reproductive success, and disease resistance. Consequently, advances in *L. vannamei* production could be significantly propelled by modulating its neuroendocrine system.

Neuropeptidomics aims to comprehensively identify the collection of neuropeptides in an organism, organ, tissue, or cell. The charting of the neuropeptidome is the first step towards understanding peptidergic signaling [9].With the advancements in RNA sequencing (RNA-seq), the application of bioinformatics methods in neuropeptide mining has been performed in various crustaceans, including *Macrobrachium nipponense* [10], *Portunus trituberculatus* [11], and *Cherax quadricarinatus* [12]. In the case of *L. vannamei*, computational transcriptome analysis combined with bioinformatics-based peptide prediction has also been utilized to expand the neuropeptidome [13,14]. Although these previous studies have added to the identification of known neuropeptides in shrimp, there are still numerous undiscovered neuropeptides or peptides with incomplete sequence information.

In crustaceans, the eyestalk X-organ/sinus gland (XO/SG) complex is the primary site for the synthesis and secretion of neuropeptides, which has been a focal point for neuropeptide research [15]. Among the identified neuropeptides, the crustacean hyperglycemic hormone (CHH) family is particularly significant, due to its multifunctional roles, which include the regulation of carbohydrate metabolism, molting, osmoregulation, and reproduction [16]. However, it is crucial to acknowledge that, despite the established functions of certain members within the CHH superfamily, their identities and functionalities remain largely uncharacterized. Additionally, although the XO/SG complex serves as a primary site for the synthesis and secretion of various significant neuropeptides, there is a paucity of information concerning other neuropeptides and their regulatory roles in relation to the CHH family in *L. vannamei*. RNA interference (RNAi) represents a powerful tool for the functional study of neuropeptides by specifically silencing their expression. Previous studies have employed RNAi to knockdown CHH expression in *L. vannamei*, leading to significant advancements in understanding their roles in controlling reproductive processes [17,18,19]. However, the overall impact of CHH silencing on the shrimp’s neuropeptidome and the subsequent physiological consequences remain to be clarified.

The elucidation of the genome now facilitates a comprehensive genome-wide analysis of neuropeptides in *L. vannamei* [20]. In this study, we performed comprehensive genome and transcriptome analyses to characterize neuropeptide-encoding genes and evaluate their expression profiles across various tissues and developmental stages. Additionally, we investigated the chromosomal localization, gene structure, and phylogenetic relationships within the CHH gene family. We also examined the expression patterns of neuropeptides in the eyestalk following the silencing of two specific CHH genes. These findings provide a foundational framework for clarifying the regulatory network between CHH and other eyestalk neuropeptides and offer a valuable resource for genetic research in *L. vannamei*.

## 2. Materials and Methods

### 2.1. Experimental Animals

Adult shrimps (mean body weight: 12.1 ± 0.4 g; mean body length: 10.0 ± 0.4 cm) and juvenile shrimps (mean body weight: 5.5 ± 0.4 g; mean body length: 7.0 ± 0.4 cm) were procured from a commercial farm in Qingdao, China. Prior to the experiment, the shrimps were subjected to a one-week acclimation period in a 300 L PVC tank. The tank was continuously supplied with aerated natural seawater, maintaining dissolved oxygen levels regularly above 6.0 mg/L. The water parameters were stringently controlled, with a salinity of 30‰, a pH of 8.0, and a temperature of 28 ± 0.5 °C. The dietary regimen included administering dry pellets thrice daily at 09:00, 15:00, and 21:00 h. Daily maintenance was carefully performed, including the removal of fecal matter and uneaten feed, using a siphon tube. Water quality was rigorously monitored, with a daily exchange of 50% of the tank volume at 09:00 h. Seawater salinity was accurately measured using an YSI multi-parameter water quality monitor (YSI Incorporated, Yellow Springs, OH, USA). All experimental procedures and animal treatments adhered strictly to the ethical guidelines established by the Animal Experiment Ethics Committee of Qingdao Agricultural University.

### 2.2. Tissue Sampling

For the purpose of in silico transcriptome mining of neuropeptides, nine adult shrimps were randomly selected. Various tissues, including the intestine, hepatopancreas, muscle, epidermis, gill, stomach, heart, hemolymph, lymphoid organ, antennal gland, eyestalk, brain ganglia, ventral nerve, and thoracic ganglia, were meticulously dissected. The excised tissues were promptly flash-frozen in liquid nitrogen and subsequently stored at −80 °C for further analysis.

### 2.3. RNA Extraction and Transcriptome Sequencing

Total RNA was extracted from the collected tissue samples utilizing the TRIzol Reagent (Vazyme, Nanjing, China). The RNA quality was evaluated using the RNA Nano 6000 Assay Kit in conjunction with the Agilent Bioanalyzer 2100 system (Agilent Technologies, Santa Clara, CA, USA), and the RNA Integrity Number (RIN) was determined. Only RNA samples that met the stringent quality criteria (OD260/OD280 = 2.0 − 2.2, OD260/OD230 ≥ 2.0, RIN ≥ 8.0, and 28S:18S ≥ 1.0) were selected for library construction. For each replicate, RNA samples from three individuals were pooled in equal amounts to create a single mixed sample. Three mixed samples were prepared for each experimental group. Illumina library preparation and sequencing were conducted by Novogene Corporation (Novogene, Beijing, China) utilizing the NovaSeq 6000 platform (Illumina, San Diego, CA, USA). Initial processing of the raw reads in FASTQ format was executed using Perl scripts. Clean reads were obtained by filtering out reads containing adapters, poly-N sequences, and low-quality reads from the raw data. Furthermore, the Q20, Q30, and GC contents of the clean data were calculated.

### 2.4. Characterization of Neuropeptides by Bioinformatics Analysis

Trinity was utilized to assemble transcriptome data from various tissues and generate unigenes. The annotation of all unigenes was conducted based on the NCBI databases, using a cut-off E-value of 1.0 × 10^−5^. To identify neuropeptides in *L. vannamei*, we performed a search in the sequence annotation file using keywords associated with known neuropeptides in other decapods. Additionally, we performed a local tblastn analysis using TBtools v2.119 software [21], employing known decapod neuropeptide precursors as query sequences [14]. Furthermore, all identified neuropeptide sequences were validated using online Blast against the genome of *L. vannamei* (GenBank number: PRJNA508983) to confirm the neuropeptide-encoding genes. The chromosomal localization and gene structure were illustrated by IBS 1.0 software [22].

The structure of the mature neuropeptides was predicted using a well-established workflow [13]. We initiated our analysis by examining the deduced precursors for the presence of signal peptides using the online tool SignalP 5.0 (https://services.healthtech.dtu.dk/services/SignalP-5.0/) (accessed on 6 November 2022). Following this, pro-hormone cleavage sites were predicted, either by adhering to the criteria established by Veenstra [23] or by aligning with recognized precursor processing schemes. Finally, the mature peptides were identified by comparing their amino acid sequences to those of known crustacean neuropeptides, ensuring a high degree of homology. Schematic diagrams of precursor structures and multiple sequence alignment of the predicted peptide sequences were generated using CLC Main Workbench v8.0 (QIAGEN, Vedbæk, Denmark). The evolutionary relationship of CHH precursors with other known orthologous precursors was analyzed, and a phylogenetic tree was constructed using MEGA X [24] through a neighbor-joining (NJ)-based approach with 1000 bootstrap trials.

### 2.5. Expression Profiles of the Neuropeptide Genes

In addition to the transcriptomic data collected from various tissues in this study, RNA-seq data from nine early developmental stages of *L. vannamei* were obtained from NCBI (SRA accession number: SRP094135). The expression profiles of neuropeptide-encoding genes were analyzed using the reference genome for *L. vannamei*. Clean reads were aligned to the reference genome using HISAT2 v2.0.5 and the mapped reads from each sample were subsequently assembled using StringTie v1.3.3 [25]. The expression abundance and variations of each gene were quantified by calculating the fragment per kilobase of transcript per million mapped reads (FPKM). The expression profiles of neuropeptide genes were visualized as a heatmap using TBtools v2.119 software [21]. In the heatmap, the transition from red to blue indicates a decrease in log_2_ (FPKM + 1) values, with red signifying high expression levels and blue denoting lower expression levels.

### 2.6. RNAi of LvCHH3 and LvVIH

To predict the target segment containing multiple functional short-interfering RNA (siRNA) sites, we utilized the online BLOCK-iT RNAi Designer (http://rnaidesigner.thermofisher.com) (accessed on 23 June 2018) to efficiently construct double-stranded RNA (dsRNA) specific to *LvCHH3* (GenBank accession number: XM_027353902.1) and *LvVIH* (GenBank accession number: KC962398.1). Primers incorporating T7 promoter sequences were designed and synthesized based on the sequences of *LvCHH3* and *LvVIH* (Table S1). The gene fragment was subsequently amplified using these T7 promoter-conjugated primers and served as a template for in vitro transcription. The dsRNAs, specifically dsCHH3 and dsVIH, were generated using the T7 RiboMAX Express RNAi System (Promega, Madison, WI, USA) in accordance with the manufacturer’s protocol. Additionally, dsGFP, targeting the green fluorescent protein (GFP), was synthesized following the same procedure.

A cohort of 45 juvenile shrimps, all in the intermolt phase, was systematically divided into 3 equal groups, each comprising 15 individuals. Each group was further subdivided into 3 replicates, with 5 shrimps per replicate. The experimental protocol involved the administration of dsCHH3, dsVIH, or dsGFP (control) via injection at a dosage of 3 μg/g body weight. After 24 h post injection, eyestalk samples were collected from a randomly selected subset of 9 shrimps per group, ensuring that 3 shrimps were taken from each replicate. The subsequent quantification of *LvCHH3* and *LvVIH* expression levels was performed using quantitative real-time PCR (qPCR) to validate the silencing of the target genes. Following this, RNA-seq was employed to examine the expression profiles of neuropeptides in the eyestalk, subsequent to CHH gene silencing.

### 2.7. Functional Analysis of Differentially Expressed Genes (DEGs)

Differential expression analysis of genes was performed between the RNAi group (dsVIH, dsCHH3) and control group (dsGFP) by DESeq2 R package (1.16.1) [26]. The resulting *p*-values were adjusted by the Benjamini–Hochberg procedure. Genes with an adjusted *p*-value < 0.05 and |log_2_(Fold Change)| > 1.0 were regarded as differentially expressed. Gene Ontology (GO) and Kyoto Encyclopedia of Genes and Genomes (KEGG) enrichment analyses of DEGs were implemented by means of the clusterProfiler R package, in which gene length bias was corrected [27]. GO terms or KEGG pathways with corrected *p*-value less than 0.05 were considered significantly enriched in a set of DEGs. To validate the Illumina sequencing data, eight DEGs were chosen for qPCR analysis by using the same RNA samples for transcriptome sequencing.

### 2.8. qPCR Analysis of Target Genes

The expression profiles of the target genes were assessed utilizing qPCR. Specific primers for both the target and reference genes are detailed in Table S1. The qPCR assays were conducted in accordance with the manufacturer’s protocol for the ChamQ Universal SYBR qPCR Master Mix Kit (Vazyme, Nanjing, China). Each reaction mixture had a total volume of 10 μL, comprising 2.0 μL of cDNA template (5 ng/μL), 5.0 μL of 2× ChamQ Universal SYBR qPCR Master Mix, 0.2 μL each of 10 μM forward and reverse primers, and 2.6 μL of RNase-free water. The qPCR assays were performed utilizing a CFX96 Touch Real-Time PCR Detection System (Bio-Rad, Hercules, CA, USA). The amplification protocol commenced with an initial pre-incubation phase at 95 °C for 30 s, succeeded by 40 cycles of denaturation at 95 °C for 10 s and annealing/extension at 60 °C for 30 s. To verify the specificity of the amplified qPCR products, melt-curve analysis was conducted. Each cDNA sample was subjected to analysis in triplicate technical replicates, while tissue samples from each experimental group were analyzed as triplicate biological replicates.

The relative expression levels of the target genes were normalized to the expression levels of the reference gene EF1α, which was quantified in each qPCR run. The relative gene expression levels were determined using the comparative Ct method and calculated via the 2^−ΔΔCt^ analysis method [28]. The qPCR data were subjected to statistical analysis using one-way ANOVA followed by Tukey’s post hoc test, conducted with SPSS version 20.0 (SPSS Inc., Chicago, IL, USA). A *p*-value of less than 0.05 was considered indicative of a statistically significant difference.

## 3. Results

### 3.1. Identification of Neuropeptide Transcript and Peptide Prediction

Forty-two cDNA libraries were generated from 14 distinct tissues of *L. vannamei* following the prescribed methodology. The raw data have been deposited in the Genome Sequence Archive in the National Genomics Data Center, China National Center for Bioinformation (GSA: CRA019665), and are publicly accessible at https://ngdc.cncb.ac.cn/gsa (accessed on 15 October 2024). Following the removal of adaptors and quality trimming, the clean reads were employed for de novo assembly, aiming to maximize the identification of neuropeptide transcripts in *L. vannamei*. Comprehensive information regarding the quality assessment, de novo assembly statistics, and annotation summary data can be found in Tables S2 and S3. Consequently, the RNA-seq analysis provided a thorough transcriptome dataset that facilitated the accurate identification of neuropeptides in *L. vannamei*.

A total of 74 putative neuropeptide precursor transcripts were identified in the assembled transcriptome. The majority of these neuropeptides had been previously identified in other crustacean and/or insect species. The mRNA sequences of newly identified precursors have been submitted to GenBank (Table 1). Furthermore, an online BLAST analysis against the reference genome of *L. vannamei* identified 125 neuropeptide-encoding genes, of which 54 had not been previously characterized (Table 1). In addition, 27 of these neuropeptide-encoding genes lacked gene ID annotations in the genome, and 4 genes were not detected within the genomic sequence.

### 3.2. The Expansion of Neuropeptide-Encoding Genes in L. vannamei

#### 3.2.1. Gonadotropin-Releasing Hormone (GnRH)-like Gene Family

**Figure 1:**
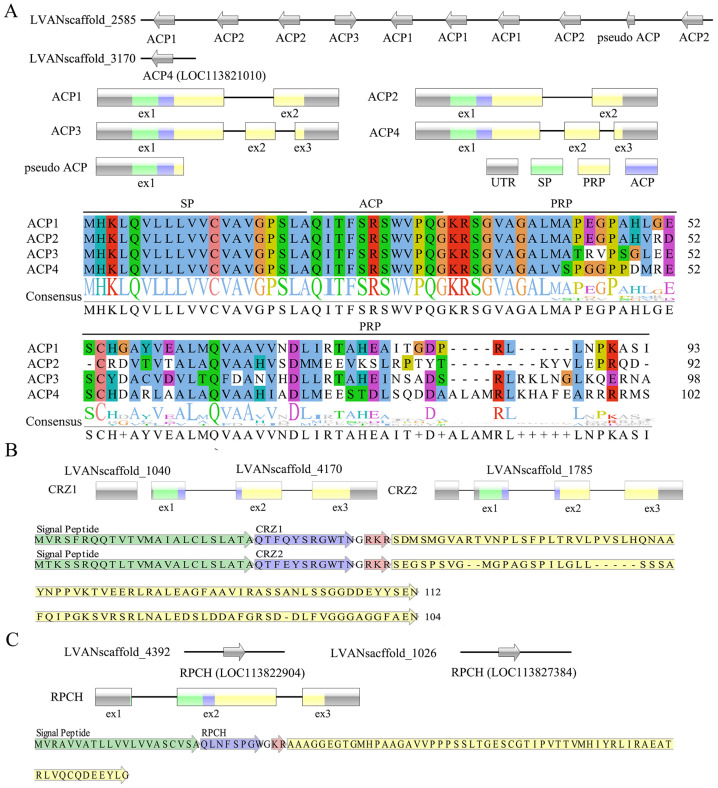
The genomic location and structural organization of GnRH-like genes, as well as the arrangement and alignment of neuropeptide precursors. (**A**) ACP; (**B**) CRZ; (**C**) RPCH. Arrows along the line denote genes located on a single scaffold. Gene structures are depicted with boxes for exons and lines for introns. Grey boxes indicate untranslated regions, green boxes denote sequences encoding single peptides (SPs), yellow boxes represent sequences encoding precursor-related peptides (PRPs), and blue boxes signify sequences encoding the mature peptides of ACP, CRZ, or RPCH. In the detailed sequences of CRZ and RPCH precursors, the mature peptides are indicated in blue, potential dibasic cleavage sites are marked in red, the SP is highlighted in green, and the PRP is shown in yellow.

In *L. vannamei*, three GnRH-like neuropeptides, namely Adipokinetic hormone/corazonin-like peptide (ACP), corazonin (CRZ), and red pigment-concentrating hormone (RPCH), were discovered. Moreover, it was observed that multiple genes were responsible for encoding the precursors of these neuropeptides (Figure 1). Specifically, four types of ACP-encoding genes were identified, with four ACP1 genes, four ACP2 genes, and one ACP3 gene arranged in tandem on one scaffold, and one ACP4 gene located on another scaffold (Figure 1A). The ACP1 and ACP2 genes were found to possess two exons, while the ACP3 and ACP4 genes exhibited three exons. The initial exon of these genes was responsible for encoding the signal peptide (SP), the mature ACP peptide of pQITFSRSWVPQamide, and a partial precursor-related peptide (PRP). Additionally, the alignment of ACP precursors demonstrated a shared consensus N-terminus, encompassing both the SP and the mature ACP peptide (Figure 1A). Two distinct CRZ precursors were encoded by two genes situated on distinct scaffolds (Figure 1B). Each gene consisted of a 5’UTR exon and three CDS exons, although the 5’UTR exon of the CRZ1 gene was located on a separate scaffold. Sequence alignment revealed that the two CRZ precursors exhibited significant similarity in their mature CRZ peptides, except for a variation at position 4 (Figure 1B). In contrast, the scenario was distinct for the RPCH precursor. Two copies of RPCH genes were identified on different scaffolds, each consisting of three exons (Figure 1C). Both genes encoded identical RPCH precursors, resulting in the production of the mature peptide QLNFSPGWamide.

#### 3.2.2. CHH Gene Superfamily

**Figure 2:**
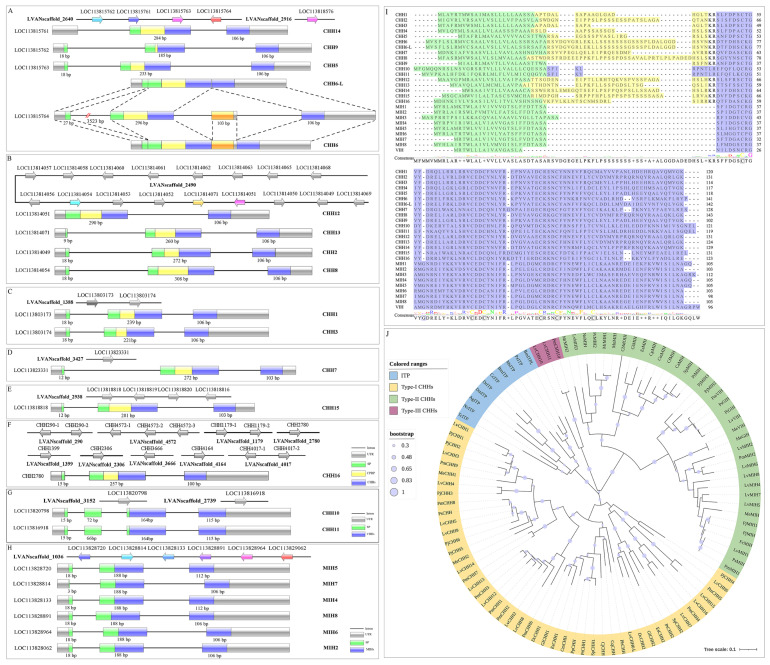
The genomic location and structural organization of CHH genes, the arrangement, alignment of CHH precursors, and phylogenetic analysis of crustacean CHH peptides and insect ITP. (**A**–**H**) Genomic location and structural organization of CHH genes; (**I**) arrangement and alignment of CHH precursors; (**J**) phylogenetic analysis of crustacean CHH peptides and insect ITP. Arrows along the line denote genes located on a single scaffold. Grey arrows represent multiple copies of the same CHH genes in a single or different scaffold. Arrows in different colors represent different CHH/MIH genes in one scaffold. Gene structures are depicted with boxes for exons and lines for introns. Grey boxes indicate untranslated regions, green boxes denote sequences encoding SP, yellow boxes represent sequences encoding CHH precursor-related peptides (CPRPs), and blue boxes signify sequences encoding the mature peptides of CHH or MIH. Specifically, the orange box is an alternative spliced exon. In the comprehensive sequences of CHH precursors, the mature peptides are denoted in blue, the SP is highlighted in green, and the CPRP is depicted in yellow. A phylogenetic tree, constructed using the MUSCLE algorithm and the NJ method implemented in MEGA X, is based on the amino acid sequences of crustacean CHH peptides and insect ITP. The node values represent the percentage derived from 1000 bootstrap replicates. The peptide sequences are detailed in Table S4.

This study has led to the identification of 52 genes within the *L. vannamei* genome that encode for CHH precursors, a significant finding that expands our understanding of the CHH family in this species. Some of the CHH genes may occur as the only gene copy on a scaffold, whereas most were organized in various tandem/duplication arrangements (Figure 2A–H). Four CHH genes were identified at scaffold 2640, with one gene encoding two precursors of CHH6 and CHH6_L through alternative splicing (Figure 2A). Additionally, one gene at scaffold 2640 encoded the precursor for CHH5, with a duplicated copy found at scaffold 2916 (Figure 2A). At scaffold 2490, a total of eighteen CHH genes were located, with fourteen of them being duplicated genes encoding the precursor for CHH2 (Figure 2B). In addition, the study revealed the presence of fourteen duplicated genes responsible for encoding the precursor for CHH16 across nine scaffolds, which had not been previously annotated in the genome (Figure 2F). Regarding the genes encoding MIH precursors, a cluster of six genes was observed in a tandem arrangement within scaffold 1036 (Figure 2H). Additionally, the gene encoding the CHH4 precursor was found to be incomplete in the genome. The genes encoding the precursors of MIH1, MIH3, and VIH were not detected in the genome in this study, despite their previous documentation and availability in the GenBank database.

The majority of CHH genes exhibit a tripartite exon organization (Figure 2A–H). The initial exon encodes a signal peptide, with the prevailing lengths being 18 or 15 base pairs. The subsequent exon decodes the signal peptide, the CHH precursor-related peptide (CPRP), which is absent in MIH genes, and a partial segment of the mature CHH peptide. The final exon encodes the remaining portion of the mature peptide, with a length of 106 base pairs being the most frequently observed.

The CHH genes encoded a total of 26 CHH/MIH/VIH precursors, as depicted in Figure 2I. Through multiple sequence alignment, it was observed that all mature peptides exhibited significant spatial conservation of six cysteines, which are distinctive features of the CHH superfamily. Furthermore, phylogenetic analysis classified crustacean CHH mature peptides into three subgroups (Figure 2J). Based on the results, the presence of CPRP distinguished Type-I CHH precursors, while Type-II CHH precursors were characterized by a Gly^12^ insertion in the mature peptides of MIH/VIH. Type-III CHH precursors displayed an intermediate structure, lacking both CPRP and the Gly^12^ insertion in the CHH10 and CHH11 precursors.

#### 3.2.3. PDH Gene Family

**Figure 3:**
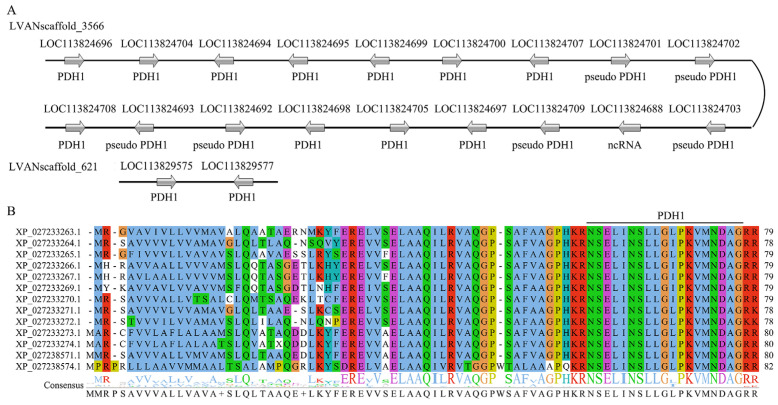
The genomic location of PDH genes and the alignment of PDH precursors. (**A**) Genomic location of PDH genes; (**B**) alignment of PDH precursors. Grey arrows along the line denote multiple copies of PDH genes located on a single scaffold.

A total of three transcripts were observed to encode putative PDH precursors, whereas a Blast analysis conducted on the *L. vannamei* genome led to the discovery of thirteen genes encoding PDH precursors (Figure 3A). Among these, eleven were found to be arranged in a tandem configuration within scaffold 3566, along with six pseudo PDH genes, while the remaining two were situated in scaffold 621. The thirteen PDH precursors were comprised of 78–82 amino acids and the C-terminal amidated PDH mature peptide was released by two dibasic cleavage sites (KR) (Figure 3B). All PDH mature peptides exhibited conserved sequence of NSELINSLLG-L/I-PKVMNDAamide (Figure 3B).

### 3.3. Alternative Splicing Creates Different Agatoxin and Calcitonin Precursors

**Figure 4:**
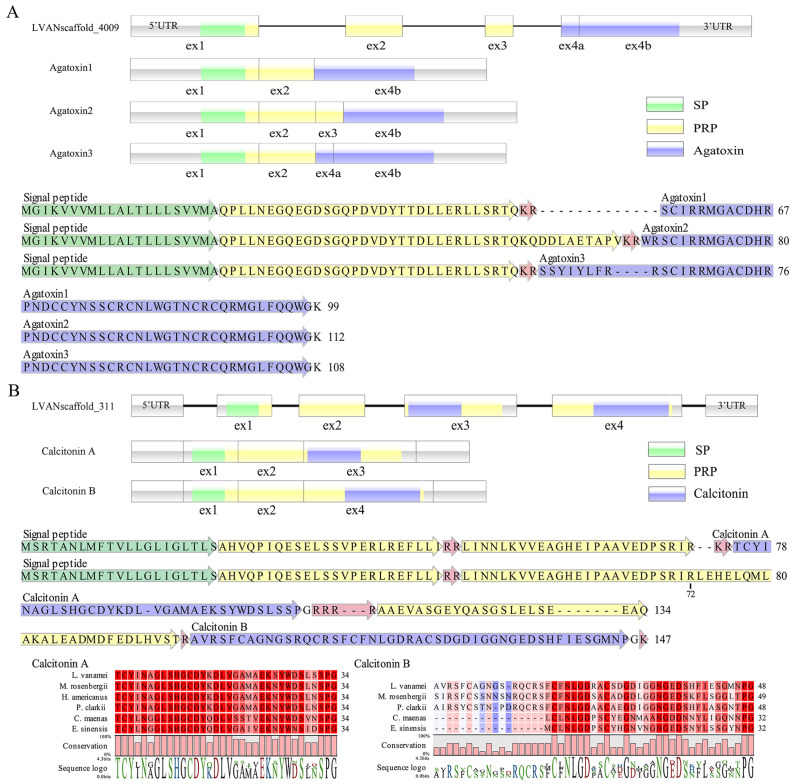
The structural organization and alternative splicing of Agatoxin and Calcitonin genes, as well as the arrangement and alignment of neuropeptides. (**A**) Agatoxin; (**B**) Calcitonin. Gene structures are depicted with boxes for exons and lines for introns. Grey boxes indicate untranslated regions, green boxes denote sequences encoding SP, yellow boxes represent sequences encoding PRP, and blue boxes signify sequences encoding the mature peptides of Agatoxin or Calcitonin. In the detailed sequences of agatoxin and calcitonin precursors, the mature peptides are indicated in blue, potential dibasic cleavage sites are marked in red, the SP is highlighted in green, and the PRP is shown in yellow. Sequences alignments of predicted Calcitonin A and Calcitonin B peptides are shown. Conserved amino acids are shown in a gradient from blue to red (blue means nearly similar, dark red means exact amino acid and white is no conservation).

Three transcripts were identified as encoding putative Agatoxin precursors, while only one gene was found in the genome. Alignment of the three Agatoxin precursors revealed that they shared the identical N-terminus and C-terminus, except for a few inserted amino acids within the precursors of Agatoxin 2 and Agatoxin 3 (Figure 4A). The Agatoxin gene consisted of four exons, with exon 4 comprising exon 4a and exon 4b. It can be inferred that the three Agatoxin precursors resulted from alternative splicing of exon 3, exon 4a, or both (Figure 4A). In a similar manner, the consensus N-terminus was shared by the precursors of Calcitonin A and Calcitonin B, while the release of two distinct types of calcitonin peptides occurred from these precursors (Figure 4B). The unique Calcitonin gene comprised four exons, and the alternative splicing of either exon 3 or exon 4 resulted in the formation of the two Calcitonin precursors. Upon sequence alignment, it was observed that both types of Calcitonin peptides possessed a predicted N-terminal disulfide bridge and a C-terminal Pro-amide sequence, except the Calcitonin B peptides in *L. vannamei*, *M. rosenbergii*, and *P*. *clarkii*, which were predicted to have two cysteine bridges at the N-terminus (Figure 4B).

### 3.4. The Identification of Other Neuropeptide Precursors

The amino acid sequences of other putative neuropeptide precursors, along with their structural information including the locations of bioactive mature peptides, cleavage sites, and precursor size, are presented in Figures S1–S4. Conserved motifs were identified in several neuropeptide families, such as AST-A (XYXFGLamide, where X represents variable residues), AST-B (XWXXXXGXWamide), Orcokinin (NFDEIDRXXXGFX), Pyrokinin (XXFXFXPRLamide), and WXXXRamide (WXXRamide).

### 3.5. Temporal- and Spatial-Expression Patterns of Neuropeptide Genes

**Figure 5:**
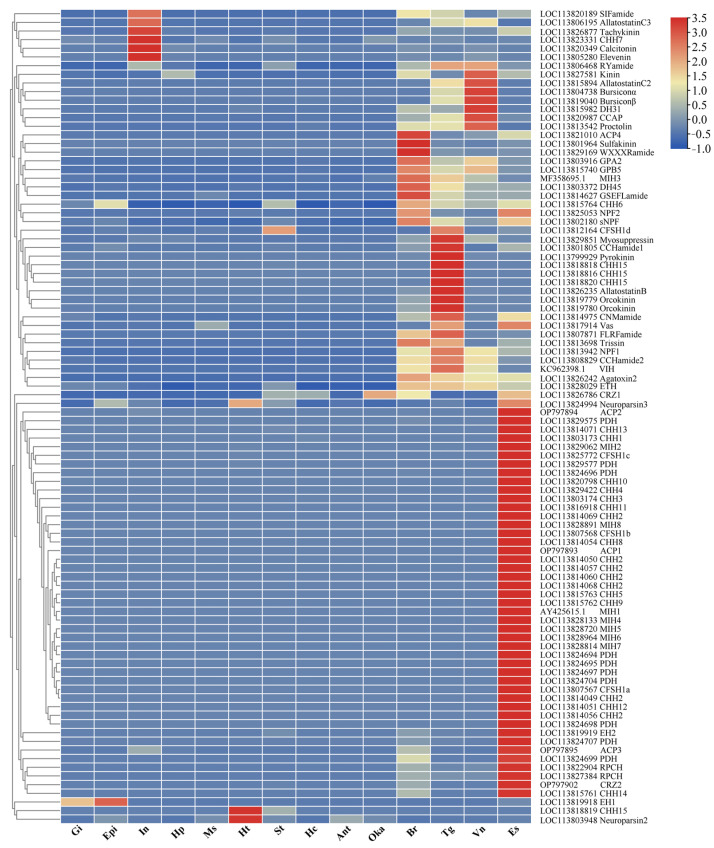
Hierarchical clustering of neuropeptide genes in various tissues. Colors represent relative mRNA expression as indicated in the color key. The red color shows high expression, and the blue color represents lower levels of expression. The color from red to blue represents the log_2_ (FPKM + 1) from large to small. Gi, gill; Epi, epidermis; In, intestine; Hp, hepatopancreas; Ms, muscle; Ht, heart; St, stomach; Hc, hemolymph; Ant, antennal gland; Oka, lymphoid organ; Br, brain ganglia; Tg, thoracic ganglia; Vn, ventral nerve; Es, eyestalk.

**Figure 6:**
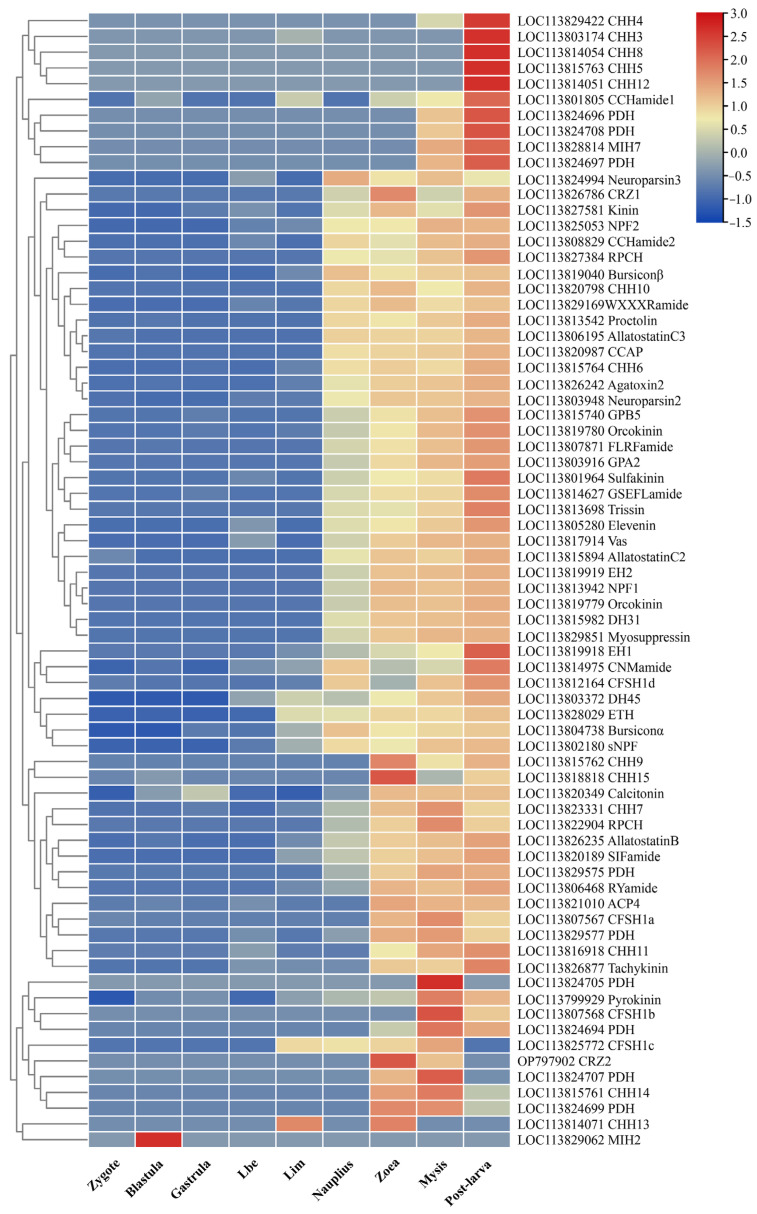
Hierarchical clustering of the expression of neuropeptide genes in various developmental stages. Colors represent relative mRNA expression, as indicated in the color key. The red color shows high expression, and the blue color represents lower levels of expression. The color from red to blue represents the log_2_ (FPKM + 1) from large to small. Lbe, limb bud embryo; Lim, larva in membrane.

An analysis was conducted to examine the temporal- and spatial-expression patterns of neuropeptide genes using transcriptomic data obtained from various tissues and developmental stages (Figure 5 and Figure 6). The results revealed that most of the neuropeptide genes exhibited distinct and elevated expression levels in neuroendocrine tissues such as the eyestalk, brain, thoracic ganglion, and ventral nerve (Figure 5). Significantly, a considerable proportion of these genes exhibited exclusive expression in the eyestalk. Notably, certain genes, such as sulfakinin, WXXXRamide, MIH3, and sNPF demonstrated elevated expression levels in the brain. Similarly, the genes AST-B, CCHamide, and orcokinin displayed distinct expression patterns in the thoracic ganglion, whereas bursicon α and bursicon β exhibited the highest expression levels in the ventral nerve. Intriguingly, we have also noted the presence of neuropeptide genes exhibiting significant expression in non-neuroendocrine tissues. For instance, genes including Calcitonin, CHH7, elevenin, and tachykinin displayed distinct expression patterns in the intestine. Similarly, one neuroparsin gene and one CHH15 gene showed highest expression levels in the heart, while the EH1 gene exhibited high levels in the gill and epidermis.

The expression of neuropeptide genes during early embryonic development was found to be minimal, except for the specific expression of the MIH2 gene in the blastula stage (Figure 6). Additionally, several genes exhibited initial expression at the larva in the membrane (Lim) stage. Furthermore, most of neuropeptide genes began to be expressed after hatching at the nauplius or zoea stage, and demonstrated high expression levels from the zoea stage to the post-larva stage. Notably, a significant proportion of these genes exhibited particularly high expression levels at the post-larva stage, such as the CHH3, CHH5, and CHH8 genes. Remarkably, the CHH9 and CHH15 displayed notably higher expression levels at the zoea stage, and the genes such as CFSH1b and pyrokinin exhibited higher expression levels at the mysis stage.

### 3.6. RNAi Efficiency of dsCHH3 and dsVIH in the Eyestalk

**Figure 7:**
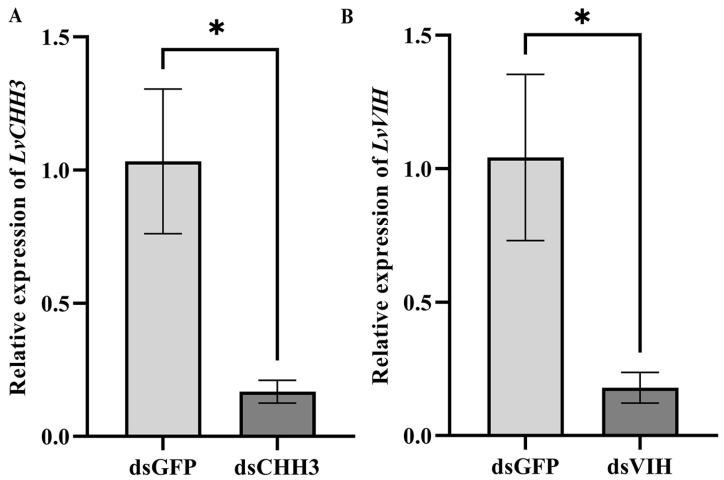
The RNAi efficiency of (**A**) dsCHH3 and (**B**) dsVIH. The results are presented as mean ± SEM, and asterisks indicate a significant difference (*p <* 0.05) between the dsGFP and dsCHH3 or dsVIH injection group.

To effectively silence the expression of *LvCHH3* and *LvVIH*, an intramuscular injection of in vitro synthesized dsRNAs (dsCHH3 and dsVIH) was meticulously administered. The subsequent analysis of *LvCHH3* and *LvVIH* expression levels in the eyestalk was conducted 24 h following the injection. The results demonstrated a successful knockdown efficacy, with an impressive 80% reduction in gene expression relative to the control group, as depicted in Figure 7.

### 3.7. Functional Analysis of DEGs in the Eyestalk After RNAi

**Figure 8:**
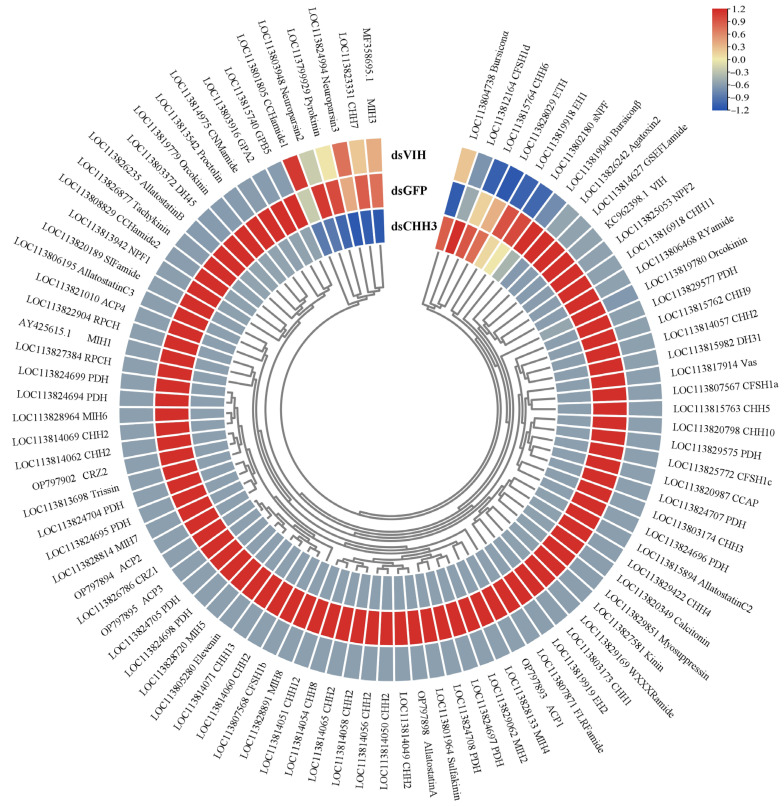
Hierarchical clustering of the expression of neuropeptide genes after the silencing of *LvCHH3* and *LvVIH*. Colors represent relative mRNA expression, as indicated in the color key. The red color shows high expression, and the blue color represents lower levels of expression. The color from red to blue represents the log_2_ (FPKM + 1) from large to small.

Nine high-quality RNA samples from eyestalks (with three replicates per group) were utilized for the construction of cDNA libraries, which were subsequently sequenced. Detailed information is provided in Table S4. The raw sequencing reads have been deposited in the SRA database (SRP200583). A significant number of genes were found to be up-regulated in dsCHH3 compared to dsGFP (61 genes), and in dsVIH compared to dsGFP (120 genes). Conversely, the number of down-regulated genes was 201 and 182, respectively. Moreover, a substantial number of the down-regulated genes encoded neuropeptides. The expression profiles of neuropeptide genes showed that the majority of the genes were down-regulated after the silencing of either *LvCHH3* or *LvVIH* (Figure 8). The results of the differential expression analysis were validated by qPCR, demonstrating the reliability and accuracy of the differential expression findings (Figure S5).

The top significantly enriched GO terms were consistent across the two comparisons (corrected *p* < 0.05; Table 2). These terms included the neuropeptide signaling pathway (GO: 0007218) and glucose metabolic process (GO: 0006006) within the biological processes category, the extracellular region (GO: 0005576) within the cellular component category, and neuropeptide hormone activity (GO: 0005184) and hormone activity (GO: 0005179) within the molecular function category. Among the enriched GO terms, the presence of “neuropeptide signaling pathway”, “neuropeptide hormone activity”, and “hormone activity” highlights the fact that the DEGs influenced by the silencing of *LvCHH3* and *LvVIH* were indeed implicated in hormonal activities. This underscored the pivotal role of these genes in processes mediated by neuropeptides.

In the two comparisons, three and five KEGG pathways were significantly enriched, respectively (corrected *p* < 0.05; Table 3). The majority of these significantly enriched pathways were categorized under metabolic pathways. In the dsGFP vs. dsCHH3 comparison, the most significantly enriched pathways included those related to amino acid metabolism, such as Phenylalanine, Tyrosine, and Tryptophan biosynthesis (ko00400), as well as Phenylalanine metabolism (ko00360). Conversely, in the dsGFP vs. dsVIH comparison, the most significantly enriched pathways pertained to lipid metabolism, specifically Arachidonic acid metabolism (ko00590). Additionally, Insect hormone biosynthesis (ko00981) was also identified as a significantly enriched pathway in the dsGFP vs. dsVIH comparison.

## 4. Discussion

### 4.1. The Expansion of Neuropeptides in L. vannamei

In this study, we conducted in silico mining of transcriptome and genome databases, to characterize the neuropeptidome of *L. vannamei*. We identified 125 neuropeptide-encoding genes, of which 54 were previously uncharacterized or unannotated. In this study, we conducted a re-annotation of neuropeptide-encoding genes (Table 1). We acknowledge the potential for annotation errors that could misrepresent gene functions and consequently distort our comprehension of the neuropeptidome in *L. vannamei*. To mitigate this risk and ensure the precision of our gene annotations, we employed a stringent annotation pipeline using NCBI databases, setting an E-value threshold of 1.0 × 10^−5^ to minimize the inclusion of false positives. Furthermore, we bolstered the accuracy of our annotations by corroborating our results with known neuropeptide sequences from other decapod species. While annotation errors may present a limitation, these rigorous measures ensure that they do not significantly undermine the overall validity of this study’s findings.

While the majority of these neuropeptides had been identified in other crustacean or insect species [14], several precursors are novel to *L. vannamei* in a broader context, such as the precursors of ACP2-ACP4 and CRZ2. The invertebrate neuropeptides, including ACP, CRZ, and RPCH, share intriguing evolutionary ties with GnRH, a neuropeptide that is widely acknowledged for its pivotal role in regulating reproduction in vertebrates [29,30]. However, the presence of GnRH in crustaceans has been a subject of debate, as only a limited number of studies have reported the presence of GnRH-like immunoreactivity [31,32], prompting the hypothesis that GnRH peptides might have been lost along the arthropod lineage [33]. Through our comprehensive and meticulous analysis, we have successfully identified all genes encoding proteins with characteristics similar to GnRH, thereby elucidating the expansion and diversification of genes encoding GnRH-like neuropeptides within the *L. vannamei* genome. This discovery significantly enhances our understanding of the evolutionary conservation and divergence of neuropeptide families, and paves the way for further exploration of their functional roles in crustacean physiology and reproduction.

The elucidation of the *L. vannamei* genome has indeed heralded a new era in our understanding of the CHH gene-family expansion. However, a comprehensive global perspective on this gene family has remained elusive [20]. The absence of three genes from the genomic sequence could be attributed to the challenges associated with genome assembly, a common limitation in genomic studies that can obscure the complete picture of gene-family representation. Our analysis of the identified CHH genes, including their structures and genomic locations, has unveiled a diverse genomic organization.

While some genes stand alone on their scaffolds, others are clustered, indicating a complex history of gene duplication and rearrangement. The hypothesis regarding the origin of the CHH superfamily members from a common ancestral gene has been a topic of interest in evolutionary biology, as suggested by previous research [34,35]. Our present study has yielded intriguing insights that have prompted us to formulate a more nuanced hypothesis regarding the evolutionary origins of the CHH superfamily in *L. vannamei*. We propose that the CHH6 gene emerges as a prime candidate for the ancestral gene within this species’ genome. This hypothesis is bolstered by several key findings. A significant observation is the structural diversity present in the C-terminus of type-I CHH peptides. Our analysis has uncovered a divergence in the C-terminus structure, where one variant is equipped with a glycine amidation site, while another is devoid of this feature. This variation is a direct outcome of the alternative splicing events occurring within the CHH6 gene, which is capable of producing these two distinct types of CHH peptides. This discovery suggests a mechanism for functional diversification and specialization within the CHH superfamily. Moreover, the CHH6 gene’s structure, which includes four exons as opposed to the three-exon configuration typical of other type-I CHH genes, lends further credence to our hypothesis. The presence of exon 3 and exon 4, both susceptible to alternative splicing, is particularly noteworthy. The coding sequences of these exons, with lengths of 103 bp and 106 bp, respectively, closely mirror the lengths of exon 3 in other type-I CHH genes (106 bp or 103 bp). This conservation in exon length between the CHH6 gene and its type-I CHH counterparts indicates a shared evolutionary heritage and provides molecular evidence of the gene duplication events that have sculpted the CHH superfamily. Additionally, the CHH6 peptide lacking the glycine amidation site has been observed for its osmoregulatory functions, akin to its homologous gene ITP found in insects [36,37]. In contrast, the type-I CHH peptide with the glycine amidation site exhibits a more pronounced role in glucose regulation, a function that appears to be unique to crustaceans [38,39]. These functional distinctions underscore the potential for the CHH6 gene to be the progenitor of the CHH superfamily in *L. vannamei*, with its alternative splicing of exon 3 potentially giving rise to a novel peptide with hyperglycemic activity. In conclusion, our study posits that the CHH6 gene could be the ancestral gene for the CHH superfamily in *L. vannamei*, highlighting the importance of alternative splicing in the generation of functionally distinct peptides.

The phenomenon of alternative splicing is not confined to the CHH superfamily, but extends to other neuropeptide precursors as well, such as agatoxin and calcitonin. Previous reports have documented the existence of three agatoxin precursors and two calcitonin precursors across various crustacean species [14]. Our study breaks new ground by unraveling the origins of these precursors for the first time, offering a window into the molecular mechanisms that have shaped the diversity of the crustacean neuropeptidome.

### 4.2. The Expression Profiles of Neuropeptide Genes

The present study represents the inaugural delineation of neuropeptide gene-expression patterns across various tissues in *L. vannamei*. Our findings highlight the fact that the majority of these genes are primarily expressed in the eyestalk, cerebral, thoracic, and ventral ganglia, consistent with their roles as neuroendocrine signaling molecules (Figure 4). Notably, a significant subset of these genes exhibited exclusive expression in the eyestalk, emphasizing the critical neuroendocrine function of the XO-SG complex [15]. A significant number of these genes are affiliated with the CHH superfamily, GnRH-like family, PDH family, and CFSH family, correlating with the XO-SG complex’s primary role in the synthesis and secretion of hormones associated with the CHH family and pigmentary-effector molecular families [40]. However, it has also been observed that numerous neuropeptides exhibit heightened expression in the cerebral, thoracic, and ventral ganglia, suggesting that these ganglia serve as significant neuroendocrine centers warranting further investigation to elucidate the regulatory roles of neuropeptides derived from these tissues. Moreover, the study identified the fact that several neuropeptide genes demonstrate peak expression levels in non-neuroendocrine tissues, including six genes in the intestine, two in the heart, and one in the epidermis. Notably, the pronounced expression of the CHH7 gene in the intestine is associated with its osmoregulatory function in *L. vannamei* [41]. These findings suggest the potential presence of endocrine cells within these tissues, which may exert autocrine effects.

Crustaceans exhibit intricate physiological mechanisms, due to the interplay of various life processes. These encompass the developmental journey from embryos to larvae, the transformative phase of metamorphosis, and the repetitive cycle of molting [42]. Transcriptomic analysis of developing embryos revealed minimal expression of neuropeptide transcripts during embryogenesis, indicating that these neuropeptide genes are not maternally expressed. Notably, an exception is observed with the MIH2 gene, which exhibits higher expression levels during the blastula stage compared to other neuropeptide genes. Furthermore, the expression of the majority of genes demonstrated a marked upregulation from the nauplius stage to the post-larva stage, which may be associated with the enhanced neuronal proliferation observed during larval development. A study investigating tachykinin-related peptide (TRP) expression across various developmental stages of *Drosophila melanogaster* demonstrated that the number of TRP-expressing neuronal cell bodies in the brain and ventral nerve cord increases during larval development, with a notable surge occurring from the later pupal stages to adulthood [43].

### 4.3. The Expression of Neuropeptide Genes After the Silencing of LvCHH3 and LvVIH

A comparative transcriptome analysis was conducted to examine the effects of CHH silencing on gene expression in the eyestalk. The results revealed a downregulation of neuropeptide genes following the suppression of either *LvCHH3* or *LvVIH*. Previous studies have demonstrated that the use of dsRNA targeting the CHH gene *Liv-SGP-G* (designated as *LvCHH5* in this study) can inhibit the transcription of not only the target gene, but also closely related CHH genes *Liv-SGP-A*, *-B*, and *-C* (designated as *LvCHH1*, *2*, and *3* in this study) in *L. vannamei* [19]. The utilization of the relative dsRNA in RNA interference may enable the co-transcriptional repression of the specific *CHH* gene, as well as other homologous *CHH* genes exhibiting high sequence similarity to the dsRNA. Nevertheless, this does not clarify the observed reduction in the expression of other neuropeptide genes.

Subsequent enrichment analysis revealed that the most significantly enriched pathways were those involved in Phenylalanine, Tyrosine, and Tryptophan biosynthesis (ko00400) and Phenylalanine metabolism (ko00360) when comparing dsGFP to dsCHH3. Tryptophan, tyrosine, and phenylalanine function as biosynthetic precursors for neurotransmitters including serotonin, dopamine, and norepinephrine [44]. The regulation of hormones from the XO/SG complex appears to be partially mediated by neurotransmitters such as dopamine, serotonin, and encephalin [45,46]. It is plausible that the silencing of *LvCHH3* affected the synthesis of monoamine neurotransmitters, subsequently influencing neuropeptide synthesis. Additionally, arachidonic acid metabolism (ko00590) was identified as the most significantly enriched pathway in the comparison between dsGFP and dsVIH. The metabolism of arachidonic acid results in its conversion into prostaglandins and thromboxane. In mammals, arachidonic acid and its derivatives play a crucial role in the release of neuropeptides within the central nervous system [47]. The findings suggest that the silencing of *LvVIH* may have disrupted arachidonic acid metabolism, thereby inhibiting neuropeptide release and subsequently repressing their expression. These findings elucidate the complex regulatory mechanisms mediated by neuropeptides and provide a foundation for future investigations into the functional roles of these critical molecules in crustacean physiology and development.

The suppression of CHH and VIH genes in the eyestalk may have far-reaching consequences for the shrimp’s physiological condition. The CHH superfamily, in particular, is known beyond its role in the eyestalk, for its involvement in regulating a range of critical functions such as carbohydrate metabolism, molting, osmoregulation, and reproduction [16]. The broader systemic effects of silencing these genes could disrupt these processes, thereby affecting the shrimp’s overall health and growth performance. Further research is essential to investigate these systemic implications and to elucidate how the downregulation of neuropeptide genes in the eyestalk can influence other physiological systems.

### 4.4. Potential Roles of Neuropeptides in Aquaculture

The implications of our findings extend beyond the realm of basic biology and into the practical applications of aquaculture. Neuropeptides, as pivotal regulators of physiological processes, present genetic targets that could revolutionize shrimp production [1]. For example, genes implicated in metabolism, molting, and reproduction, particularly those within the CHH superfamily, could be strategically manipulated to mitigate molting-related stress or to boost ovarian development [16,17,18,19]. Additionally, neuropeptides linked to stress response and disease resistance might pave the way for developing shrimp strains with enhanced resilience to environmental challenges and pathogens [48,49,50,51]. Our findings on the developmental expression patterns of neuropeptide genes also suggest that targeted interventions at critical stages could significantly improve survival rates and growth efficiency. While the prospects of neuropeptide research in aquaculture are indeed promising, there are several hurdles that need to be overcome to effectively translate these findings into tangible applications. These include navigating the complexities of neuropeptide signaling pathways and addressing the ethical concerns associated with genetic manipulation. Future research should concentrate on the functional validation of these neuropeptide genes, delve into their roles in stress response, and devise targeted interventions that can be effectively implemented within aquaculture settings.

## 5. Conclusions

In this comprehensive study, we employed a multiomics approach to systematically identify and characterize neuropeptide-encoding genes in *L. vannamei*. Certain neuropeptide-encoding gene families demonstrated significant expansion. In addition, alternative splicing was found to play a crucial role in generating functionally diverse neuropeptides. RNAi-mediated suppression of CHH and VIH genes resulted in the downregulation of the majority of neuropeptide genes. This downregulation was significantly associated with the enrichment of pathways related to amino acid metabolism and hormone synthesis, indicating the broader regulatory impact of these hormones. Our findings provide valuable genetic targets for future research aimed at improving shrimp growth and reproduction through genetic manipulation. This could lead to advancements in shrimp aquaculture practices, enhancing productivity and sustainability.

## Figures and Tables

**Table 1:** Putative neuropeptide-encoding genes predicted in *L. vannamei*.

Gene ID	Gene Description	NT ID	Neuropeptides
unannotated	uncharacterized	OP797893	Adipokinetic hormone/corazonin-like peptide 1 (ACP1)
unannotated	uncharacterized	OP797894	Adipokinetic hormone/corazonin-like peptide 2 (ACP2)
unannotated	uncharacterized	OP797895	Adipokinetic hormone/corazonin-like peptide 3 (ACP3)
LOC113821010	uncharacterized	XM_027373437.1	Adipokinetic hormone/corazonin-like peptide 4 (ACP4)
LOC113826242	U8-agatoxin-Ao1a-like	OP797896	Agatoxin1
		XM_027379116.1	Agatoxin2
		OP797897	Agatoxin3
unannotated	uncharacterized	OP797898	Allatostatin A (AST-A)
LOC113826235	prothoracicostatic peptide-like	XM_027379108.1	Allatostatin B (AST-B)
unannotated	uncharacterized	OP797899	Allatostatin C1 (AST-C1)
LOC113815894	uncharacterized	XM_027367911.1	Allatostatin C2 (AST-C2)
LOC113806195	allatostatin-like	XM_027357317.1	Allatostatin C3 (AST-C3)
LOC113804738	bursicon-like	XM_027355629.1	Bursicon α
LOC113819040	partner of bursicon-like	XM_027371277.1	Bursicon β
LOC113820349	uncharacterized	XM_027372685.1	Calcitonin A
		OP797900	Calcitonin B
LOC113801805	uncharacterized	XM_027352242.1	CCHamide1
LOC113808829	uncharacterized	XM_027360325.1	CCHamide2
unannotated	uncharacterized	OP797901	CCRFamide
LOC113814975	uncharacterized	XM_027367049.1	CNMamide
LOC113826786	uncharacterized	XM_027379674.1	Corazonin1 (CRZ1)
unannotated	uncharacterized	OP797902	Corazonin2 (CRZ2)
LOC113820987	cardioactive peptide-like	XM_027373408.1	Crustacean cardioactive peptide (CCAP)
LOC113807567	uncharacterized	XM_027358856.1	Crustacean female sex hormone1a (CFSH1a)
LOC113807568	uncharacterized	XM_027358857.1	Crustacean female sex hormone1b (CFSH1b)
LOC113825772	uncharacterized	XM_027378604.1	Crustacean female sex hormone1c (CFSH1c)
LOC113812164	uncharacterized	XM_027363996.1	Crustacean female sex hormone1d (CFSH1d)
LOC113803173	crustacean hyperglycemic hormones-like	XR_003475487.1	Crustacean hyperglycemic hormone 1 (CHH1)
LOC113814049	crustacean hyperglycemic hormones-like	XM_027366125.1	Crustacean hyperglycemic hormone 2 (CHH2)
LOC113814050		XM_027366126.1	
LOC113814052		XM_027366129.1	
LOC113814053		XM_027366130.1	
LOC113814056		XM_027366132.1	
LOC113814057		XM_027366133.1	
LOC113814058		XM_027366134.1	
LOC113814060		XM_027366135.1	
LOC113814061		XM_027366136.1	
LOC113814062		XM_027366137.1	
LOC113814063		XM_027366139.1	
LOC113814065		XM_027366140.1	
LOC113814068		XM_027366141.1	
LOC113814069		XM_027366142.1	
LOC113803174	crustacean hyperglycemic hormones	XM_027353902.1	Crustacean hyperglycemic hormone 3 (CHH3)
LOC113829422	crustacean hyperglycemic hormones 4	XM_027382596.1	Crustacean hyperglycemic hormone 4 (CHH4)
LOC113815763	crustacean hyperglycemic hormones-like	XM_027367779.1	Crustacean hyperglycemic hormone 5 (CHH5)
LOC113818576		XM_027370778.1	
LOC113815764	molt-inhibiting hormone-like	XM_027367781.1	Crustacean hyperglycemic hormone 6 (CHH6)
		XM_027367780.1	Crustacean hyperglycemic hormone 6_L (CHH6_L)
LOC113823331	crustacean hyperglycemic hormone 6-like	XM_027375947.1	Crustacean hyperglycemic hormone 7 (CHH7)
LOC113814054	crustacean hyperglycemic hormones-like	XM_027366131.1	Crustacean hyperglycemic hormone 8 (CHH8)
LOC113815762	crustacean hyperglycemic hormone	XM_027367778.1	Crustacean hyperglycemic hormone 9 (CHH9)
LOC113820798	crustacean hyperglycemic hormone-like	XM_027373159.1	Crustacean hyperglycemic hormone 10 (CHH10)
LOC113816918	uncharacterized	XM_027368909.1	Crustacean hyperglycemic hormone 11 (CHH11)
LOC113814051	crustacean hyperglycemic hormone-like	XM_027366128.1	Crustacean hyperglycemic hormone 12 (CHH12)
LOC113814071	crustacean hyperglycemic hormone-like	XM_027366143.1	Crustacean hyperglycemic hormone 13 (CHH13)
LOC113815761	crustacean hyperglycemic hormone 2-like	XM_027367777.1	Crustacean hyperglycemic hormone 14 (CHH14)
LOC113818816	molt-inhibiting hormone-like	XM_027371007.1	Crustacean hyperglycemic hormone 15 (CHH15)
LOC113818818	uncharacterized	XM_027371008.1	
LOC113818819	uncharacterized	XM_027371010.1	
LOC113818820	uncharacterized	XM_027371011.1	
unannotated	uncharacterized	OP797904	Crustacean hyperglycemic hormone 16 (CHH16)
LOC113815982	uncharacterized	XM_027368000.1	Diuretic hormone 31 (DH31)
LOC113803372	uncharacterized	XM_027354151.1	Diuretic hormone 45 (DH45)
LOC113828029	uncharacterized	XM_027380970.1	Ecdysis triggering hormone (ETH)
LOC113819918	eclosion hormone-like	XM_027372151.1	Eclosion hormone 1 (EH1)
LOC113819919	eclosion hormone-like	XM_027372154.1	Eclosion hormone 2 (EH2)
LOC113805280	uncharacterized	XM_027356262.1	Elevenin
LOC113807871	uncharacterized	XM_027359175.1	FLRFamide
LOC113803916	thyrostimulin alpha-2 subunit-like	XM_027354724.1	Glycoprotein-A2 (GPA2)
LOC113815740	thyrostimulin beta-5 subunit-like	XM_027367761.1	Glycoprotein-B5 (GPB5)
LOC113814627	PRQFV-amide-like	XM_027366675.1	GSEFLamide
LOC113827581	nucleolar protein dao-5-like	XM_027380478.1	Kinin
Not found	uncharacterized	AY425615.1	Molt-inhibiting hormone 1 (MIH1)
LOC113829062	molt-inhibiting hormone-like	XM_027382182.1	Molt-inhibiting hormone 2 (MIH2)
Not found	uncharacterized	MF358695.1	Molt-inhibiting hormone 3 (MIH3)
LOC113828133	molt-inhibiting hormone-like	XM_027381102.1	Molt-inhibiting hormone 4 (MIH4)
LOC113828720	molt-inhibiting hormone	XM_027381767.1	Molt-inhibiting hormone 5 (MIH5)
LOC113828964	molt-inhibiting hormone-like	XM_027382073.1	Molt-inhibiting hormone 6 (MIH6)
LOC113828814	molt-inhibiting hormone-like	XM_027381883.1	Molt-inhibiting hormone 7 (MIH7)
LOC113828891	molt-inhibiting hormone-like	XM_027381977.1	Molt-inhibiting hormone 8 (MIH8)
Not found	uncharacterized	KC962398.1	Vitellogenesis-inhibiting hormone (VIH)
LOC113829851	myosuppressin-like	XM_027383026.1	Myosuppressin
Not found	uncharacterized	OP797903	Neuroparsin 1 (NP1)
LOC113803948	neuroparsin-A-like	XM_027354760.1	Neuroparsin 2 (NP2)
LOC113824994	neuroparsin-A-like	XM_027377778.1	Neuroparsin 3 (NP3)
LOC113813942	neuropeptide F-like	XM_027366020.1	Neuropeptide F1 (NPF1)
LOC113825053	uncharacterized	XM_027377831.1	Neuropeptide F2 (NPF2)
LOC113802180	uncharacterized	XM_027352711.1	short Neuropeptide F (sNPF)
LOC113819779	orcokinin peptide type B-like	XM_027371972.1	Orcokinin
LOC113819780		XM_027371973.1	
LOC113824694	pigment-dispersing hormone type 1-like	XM_027377462.1	Pigment-dispersing hormone (PDH)
LOC113824695	pigment-dispersing hormone type 1-like	XM_027377463.1	
LOC113824696	pigment-dispersing hormone type 1-like	XM_027377464.1	
LOC113824697	pigment-dispersing hormone type 1-like	XM_027377465.1	
LOC113824698	pigment-dispersing hormone type 1-like	XM_027377466.1	
LOC113824699	pigment-dispersing hormone type 1-like	XM_027377468.1	
LOC113824700	pigment-dispersing hormone type 1-like	XM_027377469.1	
LOC113824704	pigment-dispersing hormone type 1-like	XM_027377470.1	
LOC113824705	pigment-dispersing hormone type 1-like	XM_027377471.1	
LOC113824707	pigment-dispersing hormone type 2-like	XM_027377472.1	
LOC113824708	pigment-dispersing hormone type 2-like	XM_027377473.1	
LOC113829575	pigment-dispersing hormone type 1-like	XM_027382770.1	
LOC113829577	pigment-dispersing hormone type 1-like	XM_027382773.1	
LOC113813542	uncharacterized	XM_027365551.1	Proctolin
LOC113799929	buccalin-like	XM_027350631.1	Pyrokinin
LOC113822904	red pigment-concentrating hormone-like	XM_027375473.1	Red pigment-concentrating hormone (RPCH)
LOC113827384	red pigment-concentrating hormone-like	XM_027380264.1	
LOC113806468	uncharacterized	XM_027357611.1	RYamide
LOC113820189	FMRFamide-related neuropeptide-like	XM_027372474.1	SIFamide
LOC113801964	uncharacterized	XM_027352452.1	Sulfakinin
LOC113826877	tachykinin-like	XM_027379777.1	Tachykinin
LOC113813698	uncharacterized	XM_027365733.1	Trissin
LOC113817914	oxytocin-neurophysin 1-like	XM_027370037.1	Vasotocin–neurophysin (VT-NP)
LOC113829169	uncharacterized	XM_027382271.1	WXXXRamide

Note: Gene ID, the Genbank accession number of the gene; Gene description, the annotation of the gene in Genbank; NT ID, the Genbank accession number of the mRNA transcribed from the gene; Neuropeptides, the neuropeptides translated from the gene. Four copies each of the ACP1 and ACP2 genes, and fourteen copies of the CHH16 gene were found in the genome.

**Table 2:** The significantly enriched GO terms.

GO Terms	Class	Description
GO:0007218	Biological Process	neuropeptide signaling pathway
GO:0006006	Biological Process	glucose metabolic process
GO:0051904	Biological Process	pigment granule transport
GO:0005576	Cellular Component	extracellular region
GO:0000235	Cellular Component	astral microtubule
GO:0038039	Cellular Component	G-protein coupled receptor heterodimeric complex
GO:0005184	Molecular Function	neuropeptide hormone activity
GO:0005179	Molecular Function	hormone activity

**Table 3:** The significantly enriched KEGG pathways.

Comparison	Pathway	KEGG Class	Pathway	*q* Value
dsGFP vs. dsCHH3	ko00400	Amino acid metabolism	Phenylalanine, tyrosine and tryptophan biosynthesis	1.79 × 10^−2^
	ko00360	Amino acid metabolism	Phenylalanine metabolism	4.32 × 10^−2^
	ko04260	Circulatory system	Cardiac muscle contraction	4.32 × 10^−2^
dsGFP vs. dsVIH	ko00590	Lipid metabolism	Arachidonic acid metabolism	7.94 × 10^−3^
	ko00981	Metabolism of terpenoids and polyketides	Insect hormone biosynthesis	2.80 × 10^−2^
	ko00910	Energy metabolism	Nitrogen metabolism	3.51 × 10^−2^
	ko00591	Lipid metabolism	Linoleic acid metabolism	3.65 × 10^−2^
	ko04976	Digestive system	Bile secretion	3.65 × 10^−2^

## Data Availability

Data are contained within the article.

## Supplementary Materials

The following supporting information can be downloaded at: https://www.mdpi.com/article/10.3390/biology13121038/s1. Table S1: Specific primers used in the current study; Table S2: Sequencing data across the 42 libraries of various tissues from *L. vannamei*; Table S3: Functional annotation of de novo transcriptome from *L. vannamei*; Table S4: The sequences of peptides used for the phylogenetic tree in Figure 2; Table S5: Summary of sequence data generated from transcriptome sequencing; Figure S1: Complete sequences from L. vannamei of the precursors containing: (A) AST-A; (B) AST-B; (C) AST-C; Figure S2: Complete sequences from L. vannamei of the precursors containing: (A) Bursicon α; (B) Bursicon β; (C) CCRFamide; (D) CNMamide; (E) CFSH; (F) DH31; (G) DH45; (H) ETH; (I) EH; (J) Elevenin; Figure S3: Complete sequences from L. vannamei of the precursors containing: (A) FLRFamide; (B) GPA2; (C) GPA5; (D) GSEFLamide; (E) Kinin; (F) Myosuppressin; (G) Neuroparsin; (H) Neuropeptide F; (I) sNPF; (J) Orcokinin; (K) Proctolin; Figure S4: Complete sequences from L. vannamei of the precursors containing: (A) Pyrokinin; (B) RYamide; (C) SIFamide; (D) Sulfakinin; (E) Tachykinin; (F) Trissin; (G) Vasotocin-neurophysin; (H) WXXXRamide; Figure S5: qPCR validation of RNA-seq data.

## Abbreviations

CHH	crustacean hyperglycemic hormone
VIH	vitellogenesis-inhibiting hormone
MIH	molt-inhibiting hormone
ACP	adipokinetic hormone/corazonin-like peptide
CRZ	corazonin
RPCH	red pigment-concentrating hormone
PDH	pigment-dispersing hormone
AST-A	allatostatin A
AST-B	allatostatin B
AST-C	allatostatin C
CCAP	crustacean cardioactive peptide
DH	diuretic hormone
ETH	ecdysis triggering hormone
EH	eclosion hormone
GPA2	glycoprotein-A2
GPB5	glycoprotein-B5
NP	neuroparsin
NPF	neuropeptide F
sNPF	short Neuropeptide F
VT-NP	aasotocin–neurophysin
