# Supplementary material for: Genome-Wide Identification and Expression of Neuropeptides and Their Expression Patterns After RNAi of CHH Genes in Pacific White Shrimp Litopenaeus vannamei
Source: Biology (Basel). 2024 Dec 11;13(12):1038. doi: 10.3390/biology13121038 (PMC11673531; doi:10.3390/biology13121038)
Supplement: Supplementary file 1 [file biology-13-01038-s001.zip › Supplementary Materials.pdf]

Table S1. Specific primers used in the current study.

Gene name	Primer type	Sequence (5'-3')	Usage
**dsVIH**	Forward	TAATACGACTCACTATAGGGCAATGAG GACCTGGCTACTGTT	dsRNA synthesis
	Reverse	TAATACGACTCACTATAGGGATTCTGG GATACTTCTTCTGCCT	
**dsCHH3**	Forward	TAATACGACTCACTATAGGGATGCTTTT GGCTTCTTTGCTG	
	Reverse	TAATACGACTCACTATAGGGCCAGTTT CCTGATAAATGGCGT	
**dsGFP**	Forward	TAATACGACTCACTATAGGGACGAAGA CCTTGCTTCTGAAG	
	Reverse	TAATACGACTCACTATAGGGAAAGGGC AGATTGTGTGGAC	
**EF1α**	Forward	TGGCTGTGAACAAGATGGAC	Expression analysis
	Reverse	AGATGGGGATGATTGGGACC	
**LvVIH**	Forward	GACGCCTTGGCTGTATTCCT	
	Reverse	CTCCCAGTCAGTTCCCTTCG	
**LvCHH3**	Forward	ATCCGATGATAGTAAAAGGCACC	
	Reverse	CAGTTTCCTGATAAATGGCGTT	
**LOC113803173**	Forward	GTTTACTTGCCGACCATCTGC	Transcriptome verification
	Reverse	CAATGAAGTGTTCCGCCAGT	
**LOC113803174**	Forward	CTGCTTCCACAACGAGGTATTC	
	Reverse	GTCTGGAGAGTCATCGCACG	
**LOC113819919**	Forward	CCAGAAAGGTCCTCCAAACG	
	Reverse	GCAGTCGGGTATGAATCGC	
**LOC113811171**	Forward	CAGGGTCTACAGTTGTTGTTTGG	
	Reverse	GGGTTCTCTGACTGTGTCTGATAAA	
**LOC113823347**	Forward	ACGCAGGTCGGGTAGTAGGT	
	Reverse	TCCCCTTTTGTTTATTGCCA	
**JQ000001.1**	Forward	CTGAAGCACCGTGGACATTG	
	Reverse	ATCGGCATTCCCCTTCGT	
**LOC113809717**	Forward	CCCTAAAGGCTTGGGATGC	
	Reverse	ATTGATATGTGTTCTGTCTGCGTAG	
**LOC113810647**	Forward	GACCGACAACCACCTCCAA	
	Reverse	TGTGGGTCAATGTCTTCAGGTC	

Table S2. Sequencing data across the 42 libraries of various tissues from *L. vannamei*

Sample	Raw reads	Clean reads	GC%	Q30%	Unigenes
Gi_1	47,973,296	46,419,106	47.96	91.69	
Gi_2	47,115,216	45,342,422	47.9	91.49	
Gi_3	47,581,522	45,609,972	48.67	91.72	
Epi_1	47,073,876	45,029,248	52.34	92.1	

Epi_2	47,639,708	45,159,114	53.72	92.08	
Epi_3	46,977,078	45,538,560	50.9	90.92	
In_1	54,843,696	53,267,020	45.75	92.49	
In_2	47,423,850	45,906,292	47.14	91.9	
In_3	46,069,850	44,603,516	47.07	90.7	
Ant_1	49,519,694	47,988,160	45.71	92.17	
Ant_2	45,349,586	44,126,302	45.25	92.01	
Ant_3	45,465,478	44,180,354	45.8	91.8	
Vn_1	46,208,106	44,649,966	47.89	91.49	
Vn_2	47,524,282	46,067,184	48.16	91.85	
Vn_3	46,629,206	44,965,436	48.83	91.89	
Hp_1	52,903,868	51,290,746	50.78	91.79	
Hp_2	47,779,364	45,999,846	50.62	92.32	
Hp_3	47,651,534	46,299,648	49.92	92.09	
Ms_1	46,145,830	44,325,434	53.49	92.17	
Ms_2	47,933,592	46,339,182	53.66	91.43	
Ms_3	55,180,690	53,013,252	53.99	92	
Oka_1	45,943,562	44,455,414	47.47	92	
Oka_2	46,057,400	44,590,440	46.66	91.2	
Oka_3	46,258,220	44,729,532	45.98	91.46	
Br_1	45,975,894	44,438,002	46.42	90.8	
Br_2	44,274,454	42,794,232	46.22	91.4	
Br_3	46,854,820	45,172,770	47.42	91.28	
St_1	45,968,720	44,118,860	48.71	90.98	
St_2	47,747,272	45,119,700	49.8	90.96	
St_3	46,540,952	45,127,814	46.98	90.85	
Ht_1	28,163,422	27,050,216	43.11	89.81	
Ht_2	28,587,550	27,666,126	43.35	90.63	
Ht_3	29,992,832	29,060,622	44.33	89.73	
Tg_1	45,321,290	44,206,898	47.72	90.89	
Tg_2	47,001,710	45,979,746	47.44	90.13	
Tg_3	42,941,986	41,870,806	47.61	90.94	
Hc_1	45,741,622	44,676,206	46.14	91.71	
Hc_2	47,094,358	45,635,512	47.12	91.72	
Hc_3	45,682,216	44,201,004	45.74	90.81	
Es_1	46,152,376	44,592,910	49.46	90.85	
Es_2	45,780,694	43,706,890	49.49	91.46	
Es_3	45,202,016	43,629,800	47.93	92.01	
Total number	1,924,272,688	1,858,944,260			100,064
Total length					107,977,413
N50 length					1,708
Mean length					1,079

Note: Clean reads denote the number of paired-end reads obtained from the cleaned data. Clean data denote the total number of the bases in the cleaned data. Q30 ratio (%) denotes the percentage of bases

of the clean data with a quality value of at least 30.

Table S3. Functional annotation of *de novo* transcriptome from *L. vannamei*

Annotated databases	Number of Unigenes	Percentage (%)
NR	35,054	35.03
NT	33,968	33.94
KO	13,493	13.48
SwissProt	21,052	21.03
PFAM	31,361	31.34
GO	31,355	31.33
KOG	12,327	12.31
Annotated in all Databases	5,438	5.43
Annotated in at least one Database	54,491	54.45
Total Unigenes	100,064	100

Table S5. Summary of sequence data generated from transcriptome sequencing

Group	Library	Raw reads	Clean reads	GC%	Q30%	Total mapping%
CTR	CTR_1	40,100,972	38,191,872	47.35	93	87.76
	CTR_2	49,878,094	47,974,604	45.81	92.85	86.38
	CTR_3	48,046,804	46,074,926	44.95	92.97	89.31
dsVIH	dsVIH_1	43,607,844	41,697,910	47.83	93.27	86.92
	dsVIH_2	46,283,476	44,400,224	46.01	92.94	87.69
	dsVIH_3	47,113,664	44,999,296	47.50	93.43	87.66
dsCHH3	dsCHH3_1	49,169,570	47,258,328	44.55	93.18	88.17
	dsCHH3_2	46,275,890	44,704,614	47.33	93.01	88.08
	dsCHH3_3	47,093,752	44,966,830	45.71	92.92	87.78

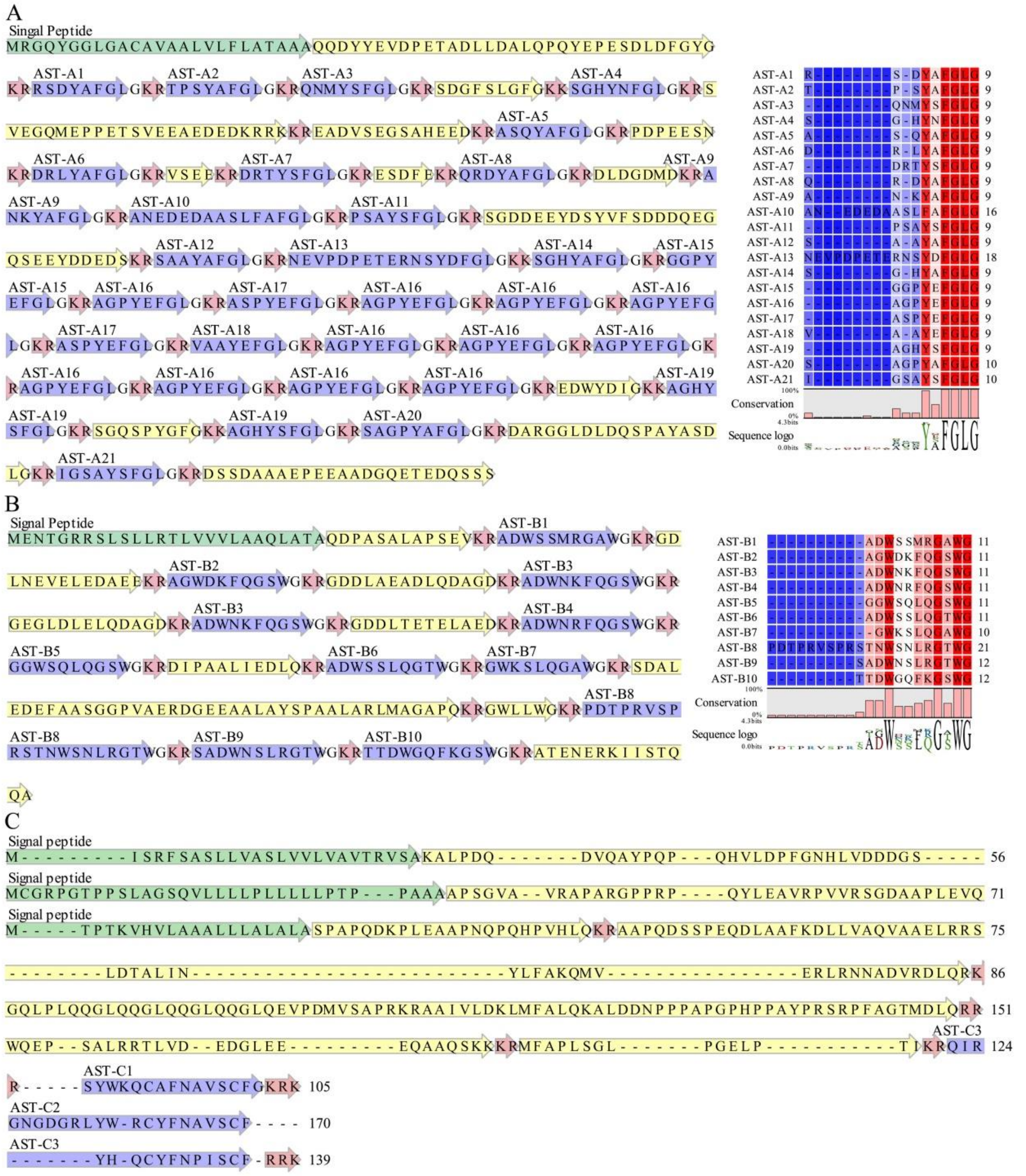

Figure S1. Complete sequences from *L. vannamei* of the precursors containing: (A) AST-A; (B) AST-B; (C) AST-C. The mature peptides are highlighted in blue, the potential dibasic cleavage sites in red, the signal peptides in green, and the PRP in yellow. Sequences alignments of predicted AST-A and AST-B peptides are shown. Conserved amino acids are shown in a gradient from blue to red (blue means nearly similar, dark red means exact amino acid and white is no conservation).

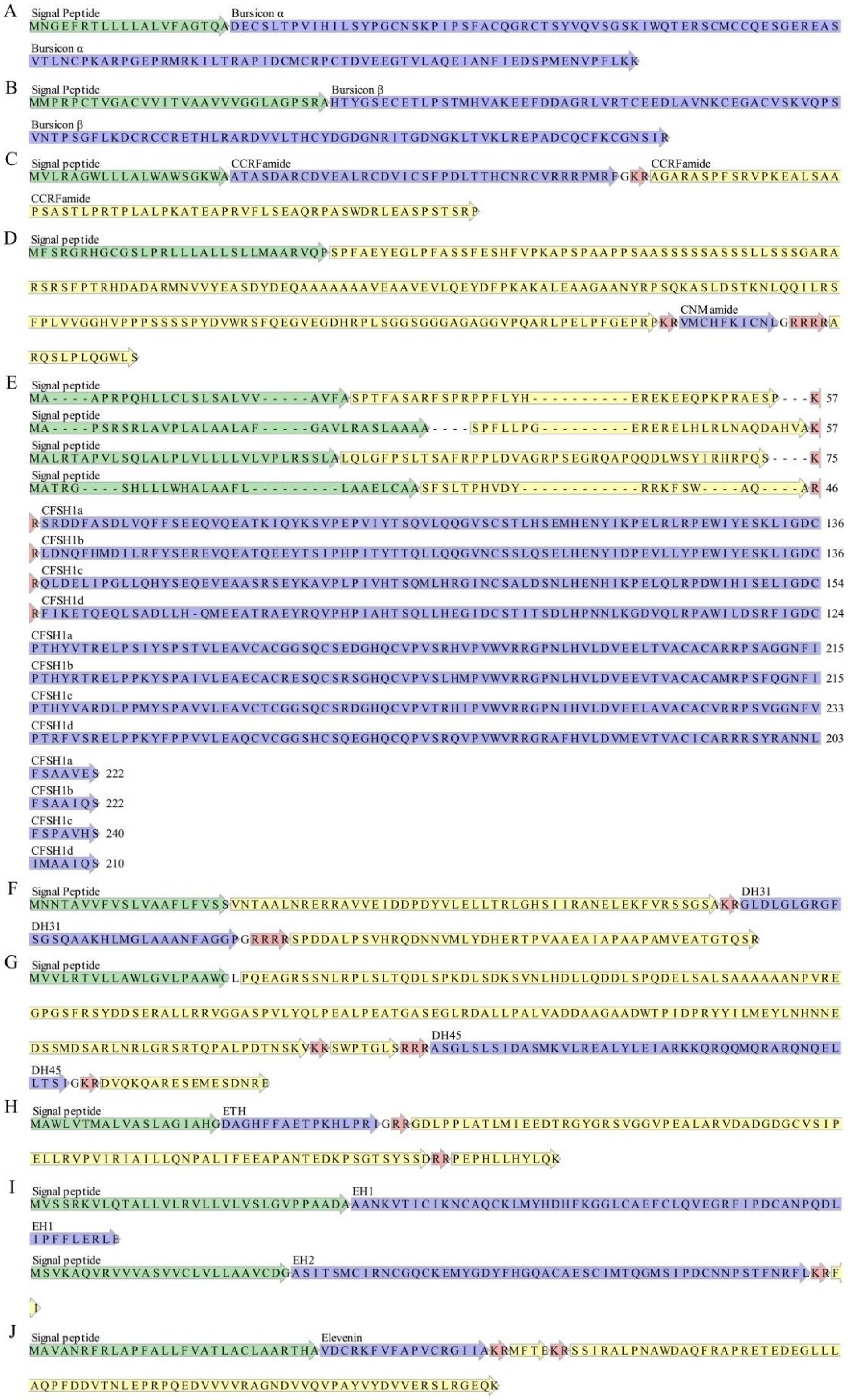

Figure S2. Complete sequences from *L. vannamei* of the precursors containing: (A) Bursicon α; (B) Bursicon β; (C) CCRFamide; (D) CNMamide; (E) CFSH; (F) DH31; (G) DH45; (H) ETH; (I) EH; (J) Elevenin. The mature peptides are highlighted in blue, the potential dibasic cleavage sites in red, the signal peptides in green, and the PRP in yellow.

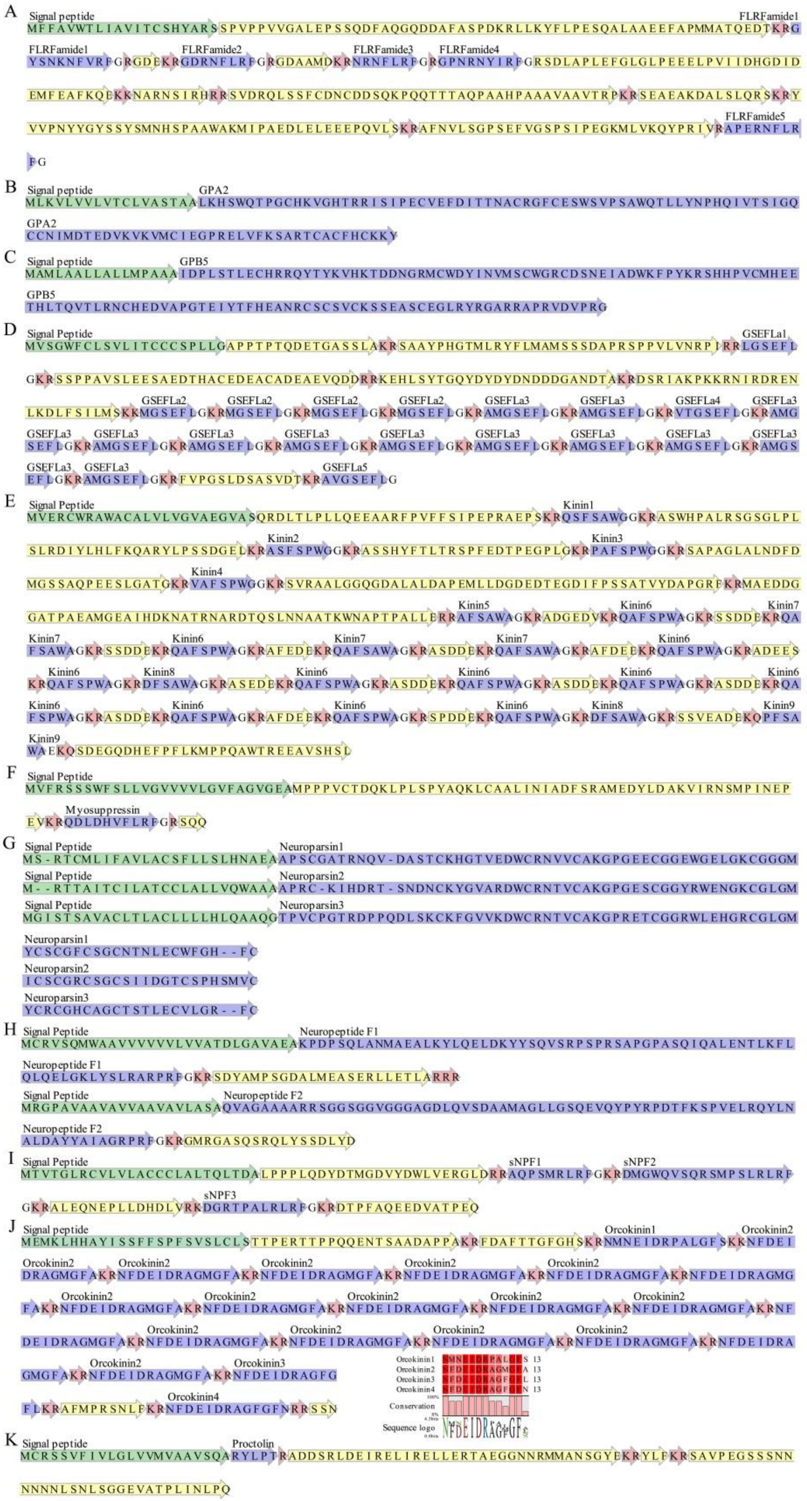

Figure S3. Complete sequences from *L. vannamei* of the precursors containing: (A) FLRFamide; (B) GPA2; (C) GPA5; (D) GSEFLamide; (E) Kinin; (F) Myosuppressin; (G) Neuroparsin; (H) Neuropeptide F; (I) sNPF; (J) Orcokinin; (K) Proctolin. The mature peptides are highlighted in blue, the potential dibasic cleavage sites in red, the signal peptides in green, and the PRP in yellow. Sequences alignments of predicted Orcokinin peptides are shown. Conserved amino acids are shown in a gradient from blue to red (blue means nearly similar, dark red means exact amino acid, and white is no conservation).

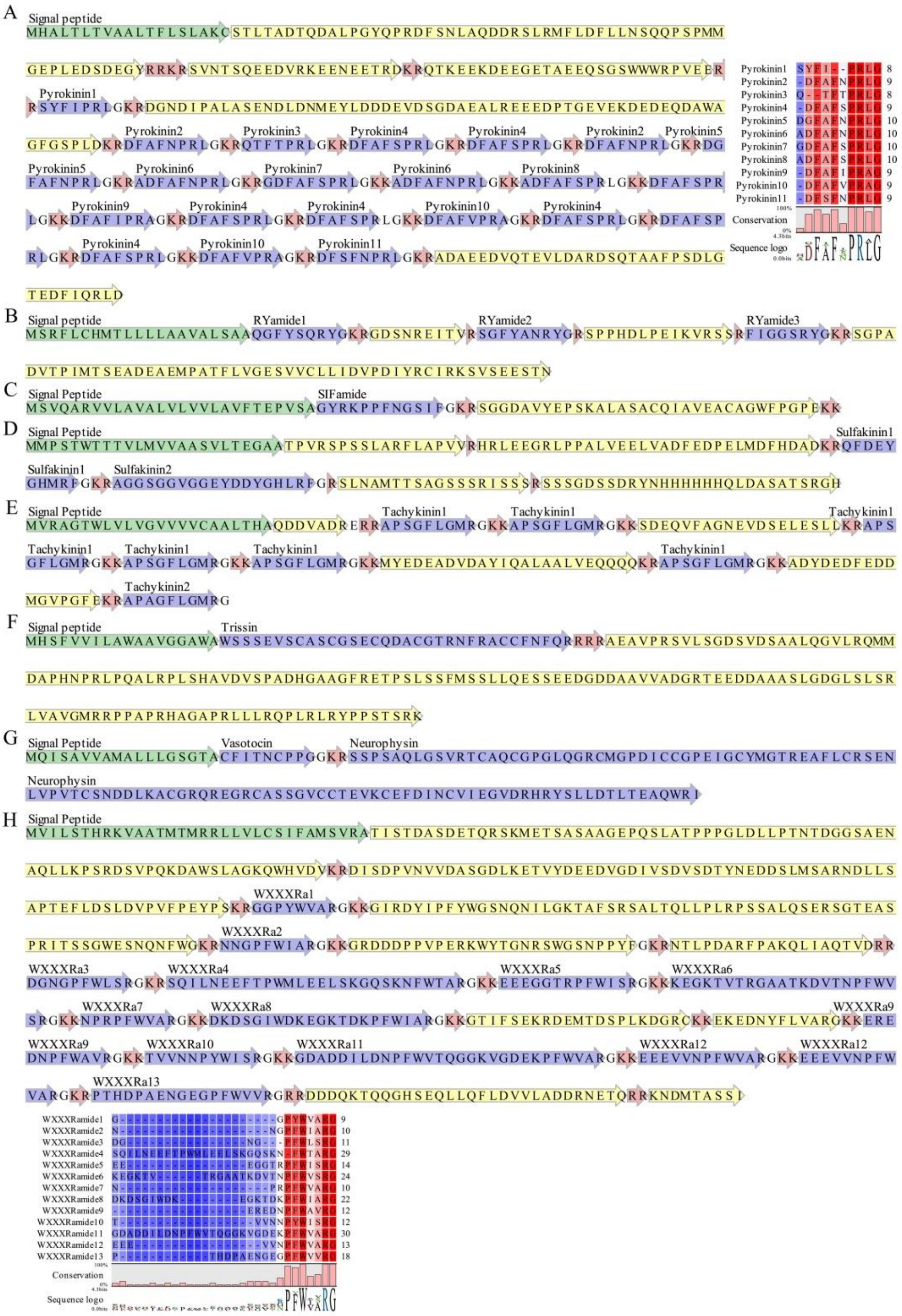

Figure S4. Complete sequences from *L. vannamei* of the precursors containing: (A) Pyrokinin; (B) RYamide; (C) SIFamide; (D) Sulfakinin; (E) Tachykinin; (F) Trissin; (G) Vasotocin-neurophysin; (H) WXXXRamide. The mature peptides are highlighted in blue, the potential dibasic cleavage sites in red, the signal peptides in green, and the PRP in yellow. Sequences alignments of predicted Pyrokinin and WXXXRamide peptides are shown. Conserved amino acids are shown in a gradient from blue to red (blue means nearly similar, dark red means exact amino acid, and white is no conservation).

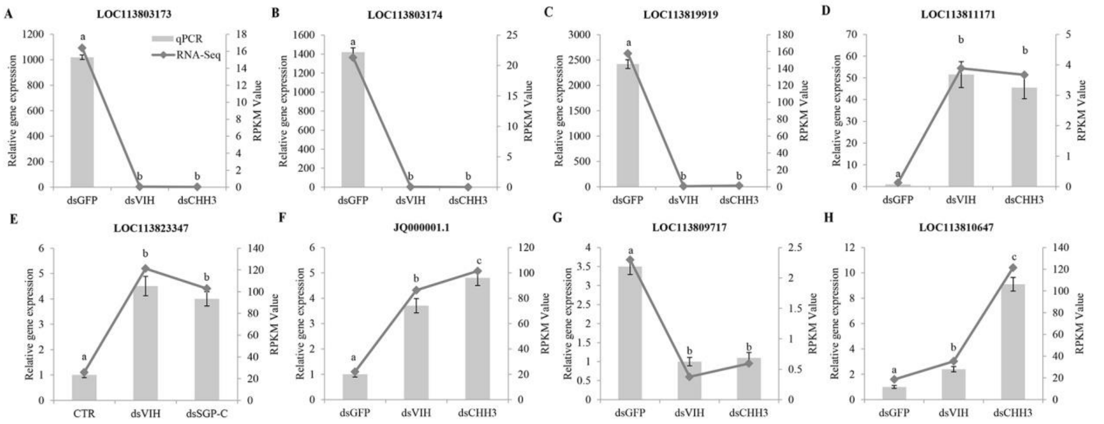

Figure S5. qPCR validation of RNA-seq data. Eight genes were selected for validation. *X* axis represents the groups. Columns and bars represent the means and standard error of relative expression levels from qPCR results (*Y* axis at left). Lines represent the FPKM value from transcriptome results (*Y* axis at right). Values with different superscripts indicated statistical significance ($p < 0.05$), which were calculated *via* one-way ANOVA
